# Supplementary material for: A generic pipeline for CADD score generation: chickenCADD and turkeyCADD
Source: G3 (Bethesda). 2025 Nov 11;16(1):jkaf272. doi: 10.1093/g3journal/jkaf272 (PMC12774583; doi:10.1093/g3journal/jkaf272)
Supplement: jkaf272_Supplementary_Data [file jkaf272_supplementary_data.docx]

**Supplementary Material**


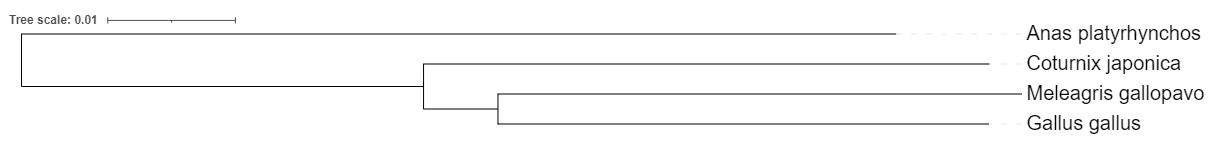


**Supplementary Figure S1.** Phylogenetic tree used for creating the 4-species multiple sequence alignment with Progressive Cactus.


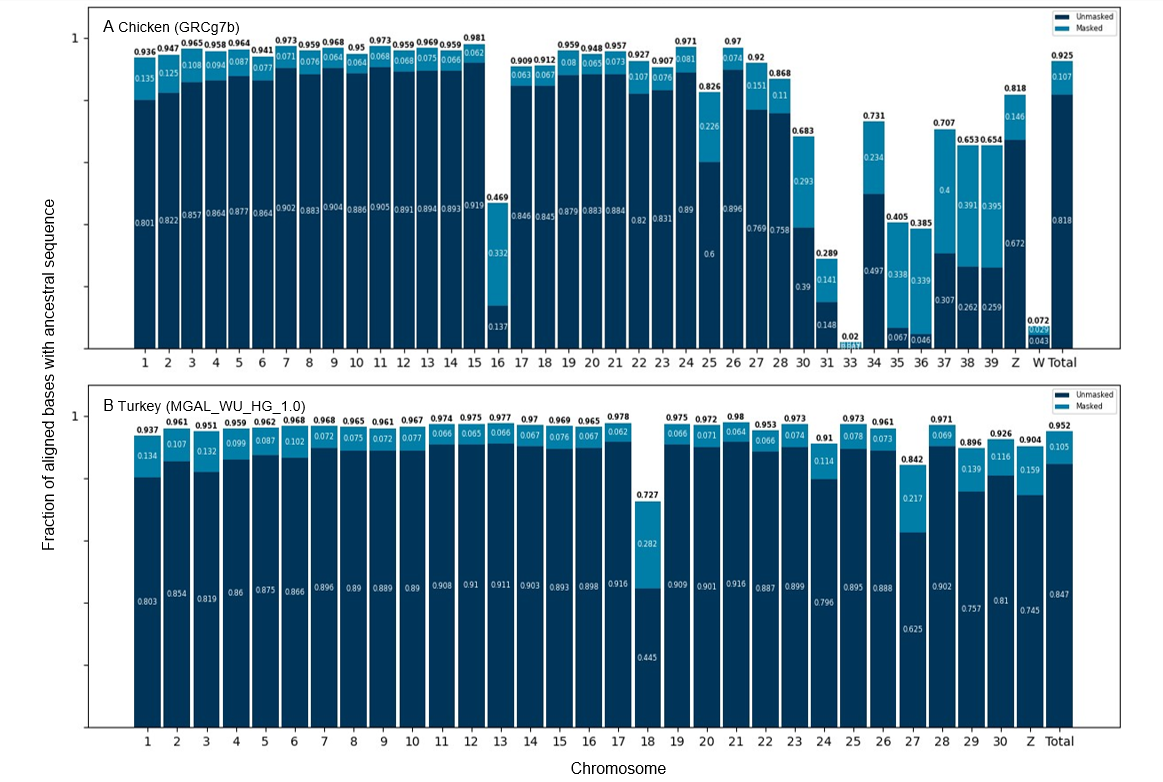


**Supplementary Figure S2.** Coverage between A. the chicken bGalGal1.mat.broiler.GRCg7b assembly and the inferred ancestral sequence and B. the turkey MGAL_WU_HG_1.0 assembly and the inferred ancestral sequence. With low coverage for the micro chromosomes.

**Supplementary Table S1.** List of annotations used for chCADD. Missing values are imputed via the specified values.

| **Annotation label** | **Data type** | **Imputed value** | **Annotation description** |
| --- | --- | --- | --- |
| Chrom | factor | - |  |
| Pos | int | - |  |
| Ref | factor | - | Reference allele |
| Alt | factor | - | Observed allele |
| isTv | bool | 0.5 | Is transversion? |
| Consequence | factor | - | VEP Consequence summaries |
| GC | num | 0.4 | Percent GC in a window of +/- 75bp |
| CpG | num | 0.02 | Percent CpG in a window of +/- 75bp |
| motifECount | int | 0 | Total number of overlapping motifs |
| motifEHIPos | factor | False | Is the position considered highly informative for an overlapping motif by VEP |
| motifEScoreChng | num | 0 | VEP score change for the overlapping motif site |
| Domain | factor | UD | Domain annotation inferred from VEP annotation (ncoils, tmhmm, sigp, lcompl, ndomain) |
| oAA | factor | UD | Amino acid of observed variant |
| nAA | factor | UD | Reference amino acid |
| Grantham | num | 0 | Grantham score: oAA,nAA |
| SIFTcat | factor | UD | SIFT category of change |
| SIFTval | num | 0 | SIFT score |
| cDNApos | num | 0 | Base position from transcription start |
| relcDNApos | num | 0 | Relative position in transcript |
| CDSpos | int | 0 | Base position from coding start |
| relCDSpos | num | 0 | Relative position in coding sequence |
| protPos | num | 0 | Amino acid position from coding start |
| relProtPos | num | 0 | Relative position in protein codon |
| dnaRoll | num | 0.2 | Predicted local DNA structure effect on dnaRoll |
| dnaProT | num | 0.5 | Predicted local DNA structure effect on dnaProT |
| dnaMGW | num | 0.01 | Predicted local DNA structure effect on dnaMGW |
| dnaHelT | num | -0.1 | Predicted local DNA structure effect on dnaHelT |
| GerpS | num | -0.3 | Rejected Substitution’ score defined by GERP++ |
| verPhyloP | num | 0.04 | 4-msa PhastCons score (excl. chicken) |
| verPhCons | num | 0.2 | 4-msa PhyloP score (excl. chicken) |
| minDistTSS | num | 100000 | Distance to closest Transcribed Sequence Start (TSS) |
| minDistTSE | num | 100000 | Distance to closest Transcribed Sequence End (TSE) |
| Chrom-muscle | factor | UD | 15 distinct chromatin states liftover from galGal6 |
| Chrom-lung | factor | UD | 15 distinct chromatin states liftover from galGal6 |
| Chrom-cortex | factor | UD | 15 distinct chromatin states liftover from galGal6 |
| Chrom-liver | factor | UD | 15 distinct chromatin states liftover from galGal6 |

**Supplementary Table S2.** List of annotations used for tCADD. Missing values are imputed via the specified values.

| **Annotation label** | **Data type** | **Imputed value** | **Annotation description** |
| --- | --- | --- | --- |
| Chrom | factor | - |  |
| Pos | int | - |  |
| Ref | factor | - | Reference allele |
| Alt | factor | - | Observed allele |
| isTv | bool | 0.5 | Is transversion? |
| Consequence | factor | - | VEP Consequence summaries |
| GC | num | 0.4 | Percent GC in a window of +/- 75bp |
| CpG | num | 0.02 | Percent CpG in a window of +/- 75bp |
| motifECount | int | 0 | Total number of overlapping motifs |
| motifEHIPos | factor | FALSE | Is the position considered highly informative for an overlapping motif by VEP |
| motifEScoreChng | factor | 0 | VEP score change for the overlapping motif site |
| Domain | factor | UD | Domain annotation inferred from VEP annotation (ncoils, tmhmm, sigp, lcompl, ndomain) |
| oAA | factor | UD | Amino acid of observed variant |
| nAA | factor | UD | Reference amino acid |
| Grantham | num | 0 | Grantham score: oAA,nAA |
| SIFTcat | factor | UD | SIFT category of change |
| SIFTval | num | 0 | SIFT score |
| cDNApos | num | 0 | Base position from transcription start |
| relcDNApos | num | 0 | Relative position in transcript |
| CDSpos | int | 0 | Base position from coding start |
| relCDSpos | num | 0 | Relative position in coding sequence |
| protPos | num | 0 | Amino acid position from coding start |
| relProtPos | num | 0 | Relative position in protein codon |
| dnaRoll | num | 0.2 | Predicted local DNA structure effect on dnaRoll |
| dnaProT | num | 0.4 | Predicted local DNA structure effect on dnaProT |
| dnaMGW | num | 0.02 | Predicted local DNA structure effect on dnaMGW |
| dnaHelT | num | -0.1 | Predicted local DNA structure effect on dnaHelT |
| GerpS | num | -0.3 | Rejected Substitution’ score defined by GERP++ liftover from Turkey_5.1 |
| verPhyloP | num | 0.04 | 4-msa PhastCons score (excl. turkey) |
| verPhCons | num | 0.2 | 4-msa PhyloP score (excl. turkey) |

**Supplementary Table S3.** VEP consequences summarized in 14 categories. If a variant has multiple annotations, the consequence is selected based on the shown hierarchy.

| **Hierarchy** | **Abbreviation** | **VEP Consequence** |
| --- | --- | --- |
| 1 | SG | Stop Gained |
| 2 | CS | Canonical Splice |
| 3 | NS | Non-Synonymous |
| 4 | SN | Synonymous |
| 5 | SL | STOP Lost |
| 6 | S | Splice Site |
| 7 | U5 | 5'-UTR |
| 8 | U3 | 3'-UTR |
| 9 | IG | Intergenic |
| 10 | NC | Noncoding-change |
| 11 | I | Intronic |
| 12 | UP | Upstream |
| 13 | DN | Downstream |
| 14 | O | Unknown / Other |

**Supplementary Table S4.** Top 10 model features in chickenCADD model with the largest assigned weight

| **Feature** | **Model weight** | **Feature explanation** |
| --- | --- | --- |
| gerpS | 0.661913 | GERP rejected substitution score |
| gerpS_Consequence_I | 0.414965 | GERP rejected substitution score for intronic sites |
| verPhCons | 0.375132 | Vertebrate PhastCons conservation score (excl. chicken) |
| verPhyloP | -0.26853 | Vertebrate PhyloP score (excl. chicken) |
| verPhCons_Consequence_I | 0.238323 | Vertebrate PhastCons conservation score (excl. chicken) for intronic sites |
| gerpS_Consequence_IG | 0.237931 | GERP rejected substitution score for intergenic sites |
| gerpS_Consequence_R | 0.231329 | GERP rejected substitution score for regulatory sites |
| verPhyloP_Consequence_I | -0.20045 | Vertebrate PhyloP score (excl. chicken) for intronic sites |
| dnaRoll | 0.175692 | Predicted local DNA structure effect on dnaRoll |
| dnaMGW | -0.16744 | Predicted local DNA structure effect on dnaMGW |

**Supplementary Table S5.** Top 10 model features in turkeyCADD model with the largest assigned weight

| **Feature** | **Model weight** | **Feature explanation** |
| --- | --- | --- |
| GerpS | 0.381656 | GERP rejected substitution score |
| verPhCons | 0.353878 | Vertebrate PhastCons conservation score (excl. turkey) |
| GerpS_Consequence_I | 0.254803 | GERP rejected substitution score for intronic sites |
| verPhCons_Consequence_I | 0.228232 | Vertebrate PhastCons conser fir vation score (excl. turkey) for intronic sites |
| GerpS_Consequence_IG | 0.18503 | GERP rejected substitution score for intergenic sites |
| SIFTval | -0.18453 | SIFT score |
| verPhCons_Consequence_IG | 0.148624 | Vertebrate PhastCons conservation score (excl. turkey) for intergenic sites |
| SIFTcat_deleterious | 0.134344 | SIFT category of change deleterious |
| verPhCons_Consequence_NS | 0.118971 | Vertebrate PhastCons conservation score (excl. turkey) for non-synonymous sites |
| GerpS_Consequence_UP | 0.090974 | GERP rejected substitution score for upstream sites |

**Supplementary Text S1.** Configuration file for chickenCADD

## config.yaml file chCADD for pipeline:https://git.wur.nl/job.vanschipstal/cadd-pipeline-v-2/-/tree/main?ref_type=heads ##

### - This workflow was developed for Snakemake 7.x.x, you may have to tweak profiles and the like for newer versions
### - Settings for the mice CADD model are used in this config as an example

## Other config files ##

# Specify location of the 2 tabular configuration files.

# Paths can either be absolute or relative to the directory in which snakemake is executed.

# The same is true for any other files in this configfile.

annotation_config:

processing: "config/annotation_processing.tsv"

interactions: "config/annotation_interactions.tsv"

## Targets ##
# Specify which outputs of the workflow are desired. (True to target, False to ignore)
# Snakemake will automatically schedule any requirements of the desired output,
# provided all settings and resources needed have been provided.
# It is recommended to follow the guide on the README to perform and validate each step one at a time.
# For updates to existing models, it makes sense to immediately skip to data preparation and check if the changes
# went as expected. A second check after model validation and then straight onwards to CADD score generation.
# targets:
# - ancestral_sequence: Extract ancestral sequence from MSA.
# - derive_variants: Derive variants based on reference, ancestral sequence and population vcf.
# - simulate_variants: Count derived variants, simulate equal number of proxy-deleterious, simulated variants.
# - generate_conservation_scores: Use PHAST toolkit to generate conservation scores from a MSA.
# - annotate_variants: Annotate all derived and simulated variants.
# - prepare_dataset: Impute missing values, one-hot-encode categorical variables and format dataset.
# - train_test_model: Train logistic regression model and test using n-fold validation.
# - validate_model: Validate model using datasets of known causal variants..
# - whole_genome_raw_scores: Annotate every possible variant and score using the trained model.
# - whole_genome_CADD: Sort and PHRED scale the raw scores.
targets:
 # Preparing dataset
 ancestral_sequence: False
 derive_variants: False
 simulate_variants: False
 generate_conservation_scores: False
 annotate_variants: False

 # Training & validating models
 prepare_dataset: False
 train_test_model: False
 validate_model: False

 # Generating CADD scores
 whole_genome_raw_scores: False
 whole_genome_CADD: True

## General configuration ##

# Which chromosomes should the model be trained on and for which should all variants be scored?

# - Train must be equal or a subset of score, it cannot contain unique chromosomes.

# - Chromosomes are expected to be in order: 1-n, X, Y, MT.

# e.g. it is possible to train on autosomal+X and then only include Y in the CADD score generation.

chromosomes:

train: [ '1','2','3','4','5','6','7','8','9','10','11','12','13','14','15','16','17','18','19','20','21','22','23','24','25','26','27','28','29', '30','31','32','33','34','35','36','37','38','39','Z','W']

score: [ '1','2','3','4','5','6','7','8','9','10','11','12','13','14','15','16','17','18','19','20','21','22','23','24','25','26','27','28','29','30','31','32','33','34','35','36','37','38','39','Z','W']

# Scientific name of species of interest, lower-case with _ instead of spaces.

species_name: "gallus_gallus"

## Alignment processing ##

# Here the input alignments are to be specified.

# Currently there are two purposes for a alignment to be processed:

# - For extracting the ancestral sequence.

# A Ensembl EPO alignment with defined ancestral sequences is expected as input.

# In general the alignments with EXTENDED do not contain ancestral sequences and are thus unsuitable.

# - For the calculation of conservation scores using PHAST.

# Any alignment with the species of interest should be usable for the phast score calculation.

# A high coverage is desirable, to in turn have good coverage for the conservation scores.

# Configuration:

# Path: Path to the folder in which the alignment files are found.

# type: should either be maf.gz or emf.gz, compressed alignments.

# MAF is recommended since the conversion from emf to MAF is slow,

# and the MAF file can also be downloaded from Ensembl directly.

# exclude_patterns: The workflow will use all files ending in type, except those including these patterns.

# Saves computation time, can be used to exclude e.g. unmapped alignments, scaffolds.

# chrom_prefix: MAF labels are expected in format Label.Chromosome.

# e.g mouse.10 but should it be mouse.chr10 specify 'chr' as prefix.

# 'None' may be specified if no prefix is present.

# filter_order: Specify the order of the species in the alignment block.

# The pipeline expects the reference to come first.

# Any species not mentioned in the order_list will be removed from the alignment.

# It matches on the full label from the start (e.g. mus will match mus_musculus and mus_spretus)

# You can add the . seperating the chr to ensure mus_musculus_BALBC doesn't match for mus_musculus.)

# clean_maf: Script that removes IUPAC uncertain nt's

# May be needed since maftools does not support them, disabled saves some time.

# pre_sorted: Should the alignment still be sorted?

# The alignment will always be split by chromosome, but it is optionally filtered by position.

# If the alignment is pre-sorted not re-sorting saves time.

alignments:

4_sp_msa:

path: "resources/alignment/4_sp_msa/"

type: "maf.gz"

exclude_patterns: [ "other", "scaffold", "epo.Y" ]

name_species_interest: "Gallus_gallus"

chrom_prefix: "None" # e.g. mus_musculus.[chr], no prefix

filter_order: "Gallus_gallus,Ancestor_Turkey_Chicken,Meleagris_gallopavo,Coturnix_japonica,Anas_platyrhynchos"

clean_maf: "False"

pre_sorted: "False"

## Deriving the ancestral sequence ##

# Specify an alignment defined above to extract the ancestral sequence from.

# This is tested on Ensembl EPO (non-extended) alignments, which are available for many species.

# ancestral_alignment: The name of the ancestral alignment in the configuration above.

# name_ancestor: In format Ancestor_[ref_species]_[alt_species], used to label the ancestor that will be extracted.
# sp1_tree_ab: The label/name of the species of interest in the evolutionary tree.
# For Ensembl this normally an abbreviated name, e.g Mmus for mus musculus, Rnor for rattus norvegicus.
# sp2_tree_ab: The label/name of the other species in the evolutionary tree.
# The inferred ancestral sequence of the last common ancestor of sp1 and sp2 in the tree will be extracted.

derive_ancestor:

ancestral_alignment: "4_sp_msa" # Must the one of the alignments defined above in Figure S1

name_ancestor: "Ancestor_chicken_turkey"

sp1_tree_ab: "Gallus_gallus" # Species 1 to generate ancestral sequence

sp2_tree_ab: "Meleagris_gallopavo" # Species 1 to generate ancestral sequence

## Variant generation ##

# Variants are generated based on the provided reference and the previously extracted ancestral sequence.

# reference_genome_wildcard: Must be path to reference genome.

# 1 file per chromosome, which is indicated by the wildcard {chr}.

# population_vcf: Population variant file, expected in vcf.gz format.

# min_ref_frequency: Minimum frequency for a variant in the population vcf to be considered.

# If above this threshold the reference nt will be replaced with the found high frequency variant,

# for the generation of derived variants.

# overestimation_factor: The Number of derived variants is multiplied by this value to get the number of simulations.

# that will be performed. Too high is inefficient, too low will yield an error, insufficient variants.

# This factor is necessary since the number of events does not exactly equate to the number of variants generated,

# and additionally some variants will be filtered out.

# Ultimately the obtained simulated variants are trimmed to obtain an equal number of derived and simulated variants.

generate_variants:

reference_genome_wildcard: "resources/genome/chr{chr}.fna"

population_vcf: "./chicken_population_vcf.vcf.gz"

derive:

min_ref_frequency: 0.9

simulate:

overestimation_factor: 2

vcf_annotation:

enabled: False

file: ""

labels:

- ""

## PHAST Conservation generation ##

# The workflow can generate phastCons and PhyloP conservation scores based on a multiple sequence alignment.

# This is done using the modified PHAST binaries from Kircher et al.

# These are able to treat the species of interest as non-informative when scoring, which the originals could not.

# Multiple configurations can be provided, for different alignments, trees and/or parameters.

# Configuration:

# alignment: The name of the alignment to be used, as defined under alignments in this configuration file.

# phast_cons_params: Specify additional configuration for phastCons.

# Input and output will be provided, here it should be chosen how the score will be generated.

# It is expected that target-coverage, expected-length and rho are given here.

# phylo_p_params: Specify additional configuration for phastCons.

# Input and output will be provided, here it should be chosen how the score will be generated.

# It is expected that the method and mode are specified, as well as any other optionals you need.

# Subtree is also allowed, though it should be noted that this is not available for phastCons.

#

# Optionals (either models or tree is required):

# tree: The phylogenetic tree to base the model on, without depth, these will be calculated.

# train_precision: PhyloFit precision, HIGH (default), MID (Regularly used) or LOW

# phast_cons_model: .mod file containing the model to be used for phastCons.

# phylo_p_model: .mod file containing the model to be used for PhyloP.

# Instead of fitting a model, based on the provided tree, a model can be specified instead.

# The same .mod file can be used for phastCons and phyloP but if downloaded from e.g. UCSC they will be different.

# When both models are present the tree or precision is not needed, since no model is trained from scratch.

phast:

vertebrate:

alignment: "4_sp_msa"

tree: "(((Gallus_gallus,Meleagris_gallopavo),Coturnix_japonica),Anas_platyrhynchos)"

phast_cons_params: "--target-coverage 0.3 --expected-length 45 --rho 0.3"

phylo_p_params: "--method=LRT --mode=CONACC"

# phylo_p_model: "resources/phast_models/"

#phast_cons_model: "resources/phast_models/"

train_precision: "HIGH"

## Ensembl API Installation ##

# The newest version of the Ensembl perl API-client was not found on conda

# therefore it is installed manually from git.

# Here you can configure the directory it is installed,

# and whether a install will be provided manually of if it should be done via my script.

# Dependencies are installed via conda.

# A gtf file should be preprocessed for use, e.g.:

# cat genomic.gtf | awk -F "\t" '$3=="exon"' | cut -f 1,4,5,7 | \

# sort -k1,1V -k2,2n -k3,3n | bgzip -c > exons.sorted.gtf.gz

ensembl-api:

directory: "resources/ensembl-api" # Without trailing /

should_install: "True" # Otherwise the ensembl perl api-client version 110 has to be installed manually.

## Basic, (reference) sequence derived annotation ##

# Provides basic columns (chrom, ref_nt, alt_nt, is_transversion)

# And sequence derived annotations: GC content, CpG.

# If a shape_file is provided, variants will also be annotated with DNAShape.

# The provided file contains the scores for all possible DNA pentamers.

basic_annotation:

include_masked: False

shape_file: "resources/annotations/pentamer_shape_scores.tsv"

## Transcript annotation ##

# If enabled, annotate variants with the distance to the closest transcription start and end sites.

# A gtf file should be preprocessed for use, e.g.:

# cat <file>.gtf | awk -F "\t" '$3=="transcript"' | cut -f 1,4,5,7 | \

# sort -k1,1V -k2,2n -k3,3n | bgzip -c > introns.sorted.gtf.gz

# tabix -s1 -b2 -e3 -f introns.sorted.gtf.gz

transcript_annotation:

enabled: True

transcript_file: "resources/annotation_file/introns.sorted.gtf.gz"

## Variant Effect Predictor ##

# VEP is used to annotate all variants with annotations like e.g. encoded aa, or SIFT score.

# Considering the amount of variants to score the species_specific cache is to be downloaded, to score offline.

# This can be done manually, or automatically by specifying the right parameters below.

vep:

cache:

directory: "resources/vep"

should_install: No # Should we install or assume that it has been installed by the user?

install_params: "--CACHE_VERSION 111 -s gallus_gallus -y bGalGal1.mat.broiler.GRCg7b" # Version, species and (optional: assembly).

exons: "resources/annotation_file/exons.gff.gz" ##not used in rule??

bed_annotation:

verPhCons:

file: "results/annotations/verPhCons.bed.gz"

should_sort: False

coord_base: 0

annotations:

verPhCons: 4

verPhyloP:

file: "results/annotations/verPhyloP.bed.gz"

should_sort: False

coord_base: 0

annotations:

verPhyloP: 4

gerpScore:

file: "results/annotations/gerpScore.bed.gz"

should_sort: False

coord_base: 0

annotations:

gerpS: 4

remap_chrom_lung:

file: "resources/chromatin_state/Lung_16_segments_chicken_liftover.bed"

should_sort: True

annotations:

lung_16_segments: 4

remap_chrom_liver:

file: "resources/chromatin_state/Liver_16_segments_chicken_liftover.bed"

should_sort: True

annotations:

liver_16_segments: 4

remap_chrom_cortex:

file: "resources/chromatin_state/Cortex_16_segments_chicken_liftover.bed"

should_sort: True

annotations:

cortex_16_segments: 4

remap_chrom_Muscle:

file: "resources/chromatin_state/Muscle_16_segments_chicken_liftover.bed"

should_sort: True

annotations:

muscle_16_segments: 4

## Basic, (reference) sequence derived annotation ##

# Provides basic columns (chrom, ref_nt, alt_nt, is_transversion)

# And sequence derived annotations: GC content, CpG.

# If a shape_file is provided, variants will also be annotated with DNAShape.

# The provided file contains the scores for all possible DNA pentamers.

basic_annotation:

include_masked: False

shape_file: "resources/pentamer_shape_scores.tsv"

## Model training ##

# The set of derived and simulated variants is split into n-folds, to create n train and test sets.
# n_folds: perform n-fold cross validation
# test_params:
# c: penalty for ridge regression
# max_iter: maximum number of iterations in fitting the model
# final_params: penalty and maximum number of iterations for the final model trained on all folds
# column_subsets: "All" will use all specified in the configuration file,
# csv files with subsets can be used to concurrently train smaller models.

model:

n_folds: 5

test_params:

c:

- 0.1

max_iter:

- 100

final_params:

c: 0.1

max_iter: 100

column_subsets:

All: "SPECIAL KEY, uses all columns"

# for the desired columns, a plot of the roc-auc score by genomic region can

# be generated. The plots will be included in the report and can be found in the results folder.

generate_roc_auc_for_cols:

- gerpS

## Validation datasets ##
# Multiple validation sets can be defined, they are processed in parallel.
# For each validation two .vcf or bgzipped vcf.gz files are expected.
# y0: Proxy neutral, e.g. common variants in the population, known neutral variants.
# y1: Proxy deleterious, e.g. known deleterious variants.
validation:

## Technical settings ##

# Here the level of parallelization can be configured.

# phast_blocks_per_split: For each chromosome, split maf into 1 file per X blocks.

# whole_genome_positions_per_file: X positions per vcf file for whole genome CADD scoring.

# Since there are three alternate nucleotides for each positions the number of variants per file is 3x this number.

parallelization:

phast_blocks_per_split: 800000

whole_genome_positions_per_file: 600000

dataset_memory_mb: 60000

**Supplementary Text S2**. Configuration file for turkeyCADD

## config.yaml file tCADD for pipeline:https://git.wur.nl/job.vanschipstal/cadd-pipeline-v-2/-/tree/main?ref_type=heads ##

### - This workflow was developed for Snakemake 7.x.x, you may have to tweak profiles and the like for newer versions
### - Settings for the mice CADD model are used in this config as an example

## Other config files ##

# Specify location of the 2 tabular configuration files.

# Paths can either be absolute or relative to the directory in which snakemake is executed.

# The same is true for any other files in this configfile.

annotation_config:

processing: "config/annotation_processing.tsv"

interactions: "config/annotation_interactions.tsv"

## Targets ##
# Specify which outputs of the workflow are desired. (True to target, False to ignore)
# Snakemake will automatically schedule any requirements of the desired output,
# provided all settings and resources needed have been provided.
# It is recommended to follow the guide on the README to perform and validate each step one at a time.
# For updates to existing models, it makes sense to immediately skip to data preparation and check if the changes
# went as expected. A second check after model validation and then straight onwards to CADD score generation.
# targets:
# - ancestral_sequence: Extract ancestral sequence from MSA.
# - derive_variants: Derive variants based on reference, ancestral sequence and population vcf.
# - simulate_variants: Count derived variants, simulate equal number of proxy-deleterious, simulated variants.
# - generate_conservation_scores: Use PHAST toolkit to generate conservation scores from a MSA.
# - annotate_variants: Annotate all derived and simulated variants.
# - prepare_dataset: Impute missing values, one-hot-encode categorical variables and format dataset.
# - train_test_model: Train logistic regression model and test using n-fold validation.
# - validate_model: Validate model using datasets of known causal variants..
# - whole_genome_raw_scores: Annotate every possible variant and score using the trained model.
# - whole_genome_CADD: Sort and PHRED scale the raw scores.
targets:
 # Preparing dataset
 ancestral_sequence: False
 derive_variants: False
 simulate_variants: False
 generate_conservation_scores: False
 annotate_variants: False

 # Training & validating models
 prepare_dataset: False
 train_test_model: False
 validate_model: False

 # Generating CADD scores
 whole_genome_raw_scores: False
 whole_genome_CADD: True

## General configuration ##

# Which chromosomes should the model be trained on and for which should all variants be scored?

# - Train must be equal or a subset of score, it cannot contain unique chromosomes.

# - Chromosomes are expected to be in order: 1-n, X, Y, MT.

# e.g. it is possible to train on autosomal+X and then only include Y in the CADD score generation.

chromosomes:

train: [ '1','2','3','4','5','6','7','8','9','10','11','12','13','14','15','16','17', '18', '19','20','21','22','23','24','25','26','27','28','29','30','31','32','33','34','35','Z' ]

score: [ '1','2','3','4','5','6','7','8','9','10','11','12','13','14','15','16','17', '18', '19','20','21','22','23','24','25','26','27','28','29','30','31','32','33','34','35','Z' ]

# Scientific name of species of interest, lower-case with _ instead of spaces.

species_name: "Meleagris_gallopavo"

## Alignment processing ##

# Here the input alignments are to be specified.

# Currently there are two purposes for a alignment to be processed:

# - For extracting the ancestral sequence.

# A Ensembl EPO alignment with defined ancestral sequences is expected as input.

# In general the alignments with EXTENDED do not contain ancestral sequences and are thus unsuitable.

# - For the calculation of conservation scores using PHAST.

# Any alignment with the species of interest should be usable for the phast score calculation.

# A high coverage is desirable, to in turn have good coverage for the conservation scores.

# Configuration:

# Path: Path to the folder in which the alignment files are found.

# type: should either be maf.gz or emf.gz, compressed alignments.

# MAF is recommended since the conversion from emf to MAF is slow,

# and the MAF file can also be downloaded from Ensembl directly.

# exclude_patterns: The workflow will use all files ending in type, except those including these patterns.

# Saves computation time, can be used to exclude e.g. unmapped alignments, scaffolds.

# chrom_prefix: MAF labels are expected in format Label.Chromosome.

# e.g mouse.10 but should it be mouse.chr10 specify 'chr' as prefix.

# 'None' may be specified if no prefix is present.

# filter_order: Specify the order of the species in the alignment block.

# The pipeline expects the reference to come first.

# Any species not mentioned in the order_list will be removed from the alignment.

# It matches on the full label from the start (e.g. mus will match mus_musculus and mus_spretus)

# You can add the . seperating the chr to ensure mus_musculus_BALBC doesn't match for mus_musculus.)

# clean_maf: Script that removes IUPAC uncertain nt's

# May be needed since maftools does not support them, disabled saves some time.

# pre_sorted: Should the alignment still be sorted?

# The alignment will always be split by chromosome, but it is optionally filtered by position.

# If the alignment is pre-sorted not re-sorting saves time.

alignments:

4_sp_msa:

path: "resources/alignment/4_sp_msa/"

type: "maf.gz"

exclude_patterns: [ "other", "scaffold", "epo.Y" ]

name_species_interest: "Meleagris_gallopavo"

chrom_prefix: "None" # mus_musculus.[chr], no prefix

filter_order: "Meleagris_gallopavo,Gallus_gallus,Coturnix_japonica,Anas_platyrhynchos"

clean_maf: "False"

pre_sorted: "False"

## Deriving the ancestral sequence ##

# Specify an alignment defined above to extract the ancestral sequence from.

# This is tested on Ensembl EPO (non-extended) alignments, which are available for many species.

# ancestral_alignment: The name of the ancestral alignment in the configuration above.

# name_ancestor: In format Ancestor_[ref_species]_[alt_species], used to label the ancestor that will be extracted.
# sp1_tree_ab: The label/name of the species of interest in the evolutionary tree.
# For Ensembl this normally an abbreviated name, e.g Mmus for mus musculus, Rnor for rattus norvegicus.
# sp2_tree_ab: The label/name of the other species in the evolutionary tree.
# The inferred ancestral sequence of the last common ancestor of sp1 and sp2 in the tree will be extracted.

derive_ancestor:

ancestral_alignment: "4_sp_msa" # Must the one of the alignments defined above in Figure S1

name_ancestor: "Ancestor_turkey_chicken"

sp1_tree_ab: "Meleagris_gallopavo" # Species 1 to generate ancestral sequence

sp2_tree_ab: "Gallus_gallus" # Species 2 to generate ancestral sequence

## Variant generation ##

# Variants are generated based on the provided reference and the previously extracted ancestral sequence.

# reference_genome_wildcard: Must be path to reference genome.

# 1 file per chromosome, which is indicated by the wildcard {chr}.

# population_vcf: Population variant file, expected in vcf.gz format.

# min_ref_frequency: Minimum frequency for a variant in the population vcf to be considered.

# If above this threshold the reference nt will be replaced with the found high frequency variant,

# for the generation of derived variants.

# overestimation_factor: The Number of derived variants is multiplied by this value to get the number of simulations.

# that will be performed. Too high is inefficient, too low will yield an error, insufficient variants.

# This factor is necessary since the number of events does not exactly equate to the number of variants generated,

# and additionally some variants will be filtered out.

# Ultimately the obtained simulated variants are trimmed to obtain an equal number of derived and simulated variants.

generate_variants:

reference_genome_wildcard: "./resources/genome/chr{chr}.fna"

population_vcf: "Turkey_population_vcf.vcf.gz"

derive:

min_ref_frequency: 0.9

simulate:

overestimation_factor: 2

## PHAST Conservation generation ##

# The workflow can generate phastCons and PhyloP conservation scores based on a multiple sequence alignment.

# This is done using the modified PHAST binaries from Kircher et al.

# These are able to treat the species of interest as non-informative when scoring, which the originals could not.

# Multiple configurations can be provided, for different alignments, trees and/or parameters.

# Configuration:

# alignment: The name of the alignment to be used, as defined under alignments in this configuration file.

# phast_cons_params: Specify additional configuration for phastCons.

# Input and output will be provided, here it should be chosen how the score will be generated.

# It is expected that target-coverage, expected-length and rho are given here.

# phylo_p_params: Specify additional configuration for phastCons.

# Input and output will be provided, here it should be chosen how the score will be generated.

# It is expected that the method and mode are specified, as well as any other optionals you need.

# Subtree is also allowed, though it should be noted that this is not available for phastCons.

#

# Optionals (either models or tree is required):

# tree: The phylogenetic tree to base the model on, without depth, these will be calculated.

# train_precision: PhyloFit precision, HIGH (default), MID (Regularly used) or LOW

# phast_cons_model: .mod file containing the model to be used for phastCons.

# phylo_p_model: .mod file containing the model to be used for PhyloP.

# Instead of fitting a model, based on the provided tree, a model can be specified instead.

# The same .mod file can be used for phastCons and phyloP but if downloaded from e.g. UCSC they will be different.

# When both models are present the tree or precision is not needed, since no model is trained from scratch.

phast:

vertebrate:

alignment: "4_sp_msa"

tree: "(((Gallus_gallus,Meleagris_gallopavo),Coturnix_japonica),Anas_platyrhynchos)"

phast_cons_params: "--target-coverage 0.3 --expected-length 45 --rho 0.3"

#phast_cons_model: "resources/phast_models/"

phylo_p_params: "--method=LRT --mode=CONACC"

# phylo_p_model: "resources/phast_models/"

train_precision: "HIGH"

## Ensembl API Installation ##

# The newest version of the Ensembl perl API-client was not found on conda

# therefore it is installed manually from git.

# Here you can configure the directory it is installed,

# and whether a install will be provided manually of if it should be done via my script.

# Dependencies are installed via conda.

ensembl-api:

directory: "resources/ensembl-api" # Without trailing /

should_install: "True" # Otherwise the ensembl perl api-client version 110 has to be installed manually.

## Variant Effect Predictor ##

# VEP is used to annotate all variants with annotations like e.g. encoded aa, or SIFT score.

# Considering the amount of variants to score the species_specific cache is to be downloaded, to score offline.

# This can be done manually, or automatically by specifying the right parameters below.

#vep:

# cache:

# directory: "resources/vep"

## Basic, (reference) sequence derived annotation ##

# Provides basic columns (chrom, ref_nt, alt_nt, is_transversion)

# And sequence derived annotations: GC content, CpG.

# If a shape_file is provided, variants will also be annotated with DNAShape.

# The provided file contains the scores for all possible DNA pentamers.

basic_annotation:

include_masked: False

shape_file: "resources/pentamer_shape_scores.tsv"

## Transcript annotation ##

# If enabled, annotate variants with the distance to the closest transcription start and end sites.

# A gtf file should be preprocessed for use, e.g.:

# cat <file>.gtf | awk -F "\t" '$3=="transcript"' | cut -f 1,4,5,7 | \

# sort -k1,1V -k2,2n -k3,3n | bgzip -c > introns.sorted.gtf.gz

# tabix -s1 -b2 -e3 -f introns.sorted.gtf.gz

transcript_annotation:

enabled: True

transcript_file: "resources/annotation_file/introns.sorted.gff.gz"

## VCF file based annotation ##

# Accepts a sorted, tabix indexed, bgzip compressed vcf file with annotations

# As INFO fields. Only the labels specified here will be used.

# Annotation is done via bcftools, a valid header is required.

# The tag datatype is not supported.

# enabled: should a vcf annotation be included?

# file: the .vcf.gz to take the annotation from

# label: list of annotation labels to use. Must be the same as in the vcf file.

vcf_annotation:

enabled: False

file: ""

labels:

- ""

bed_annotation:

verPhCons:

file: "results/annotations/vertebratePhastCons.bed.gz"

should_sort: False

annotations:

verPhCons: 4

verPhyloP:

file: "results/annotations/vertebratePhyloP.bed.gz"

should_sort: False

annotations:

verPhyloP: 4

gerpScore:

file: "results/annotations/gerpScore.bed.gz"

should_sort: False

annotations:

gerpS: 4

## Model training ##

# The set of derived and simulated variants is split into n-folds, to create n train and test sets.
# n_folds: perform n-fold cross validation
# test_params:
# c: penalty for ridge regression
# max_iter: maximum number of iterations in fitting the model
# final_params: penalty and maximum number of iterations for the final model trained on all folds
# column_subsets: "All" will use all specified in the configuration file,
# csv files with subsets can be used to concurrently train smaller models.

model:

n_folds: 5

test_params:

c:

- 0.1

- 10.0

max_iter:

- 100

final_params:

c: 0.1

max_iter: 100

# for the desired columns, a plot of the roc-auc score by genomic region can
# be generated. The plots will be included in the report and can be found in the results folder.

generate_roc_auc_for_cols:

- gerpS

- verPhyloP

- verPhCons

## Validation datasets ##
# Multiple validation sets can be defined, they are processed in parallel.
# For each validation two .vcf or bgzipped vcf.gz files are expected.
# y0: Proxy neutral, e.g. common variants in the population, known neutral variants.
# y1: Proxy deleterious, e.g. known deleterious variants.
validation:

## Technical settings ##

# Here the level of parallelization can be configured.

# phast_blocks_per_split: For each chromosome, split maf into 1 file per X blocks.

# whole_genome_positions_per_file: X positions per vcf file for whole genome CADD scoring.

# Since there are three alternate nucleotides for each positions the number of variants per file is 3x this number.

parallelization:

phast_blocks_per_split: 800000

whole_genome_positions_per_file: 600000

dataset_memory_mb: 60000
